# Impact of Total Ischemic Time on the Recovery of Regional Wall Motion Abnormality after STEMI in the Modern Reperfusion Era

**DOI:** 10.1155/2022/2447707

**Published:** 2022-01-22

**Authors:** Jeong Hun Seo, Kang Hee Kim, Kwang-Jin Chun, Bong-Ki Lee, Byung-Ryul Cho, Dong Ryeol Ryu

**Affiliations:** ^1^Division of Cardiology, Department of Internal Medicine, Kangwon National University Hospital, Kangwon National University School of Medicine, Chuncheon-Si, Gangwon-Do, Republic of Korea; ^2^Department of Internal Medicine, Kangwon National University Hospital, Chuncheon-Si, Gangwon-Do, Republic of Korea

## Abstract

**Background:**

Total ischemic time (TIT) is an important factor for predicting mortality among patients with ST-segment elevation myocardial infarction (STEMI). However, the correlation between TIT and the extent of wall motion abnormality has not been well studied. Therefore, we investigated changes in the wall motion score index (WMSI) value based on TIT in STEMI patients who underwent primary percutaneous coronary intervention (PCI) and subsequent transthoracic echocardiography.

**Methods:**

STEMI patients who underwent primary PCI and follow-up coronary angiography were analyzed after the exclusion of cases of in-stent restenosis (ISR). WMSI values were calculated by dividing the sum of scores by the number of segments visualized.

**Results:**

A total of 189 patients underwent primary PCI for STEMI, and 151 had no ISR with a median follow-up of 12.3 months. TIT was 180 (117–369) minutes in a subset of 151 patients (mean age of 62 years; 76% male). Among patients without ISR, 109 (72%) demonstrated a decrease in the WMSI value during the follow-up period. The WMSI values of patients with TITs of 180 minutes or less were significantly decreased relative to those among patients with TITs of greater than 180 minutes (*p*=0.020). Among patients with TITs of 180 minutes or less, the TIT was significantly shorter among those with a reduction in the WMSI value than among those with an increase in the WMSI value (106 [81–124] vs. 133 [100–151] minutes; *p*=0.018). TIT was an independent predictor for a reduction in the WMSI value among these patients (adjusted hazard ratio: 0.976 (0.957–0.995); *p*=0.016).

**Conclusions:**

In the modern reperfusion era of STEMI, patients with TITs of 180 minutes or less experienced a significant degree of recovery from regional wall motion abnormalities.

## 1. Introduction

Timely primary percutaneous coronary intervention (PCI) is the current preferred strategy to treat ST-segment elevation myocardial infarction (STEMI) [1]. The maximum myocardial salvage gained from reperfusion therapy is generally accepted to occur within the first few hours of symptom onset, and the potential for salvaging myocardium is considered minimal or absent after this time. [2, 3]. Although the data remain somewhat controversial, the bulk of the evidence also suggests that prolonged total ischemic times (TITs), times from symptom onset to balloon inflation, are associated with an increased risk of mortality [4, 5].

The longer the artery is occluded, the more the wavefront of ischemia extends radially from the endocardium to the epicardium [6]. Importantly, the transmural extent and overall size of the infarction are independently predictive of cardiac prognosis in the longer term [7]. Several studies have provided evidence that primary PCI improves left ventricular (LV) dysfunction and inhibits postinfarction dilation and remodeling [8, 9]. However, little data are available concerning the correlation between TIT and the extent of wall motion abnormality. In addition, although shorter TITs are better, the limitations of the optimal TIT are unclear. Therefore, we sought to investigate changes in the wall motion score index (WMSI) value based on the TIT in STEMI patients who underwent primary PCI and subsequent transthoracic echocardiography (TTE).

## 2. Methods

### 2.1. Study Population

**Figure 1:**
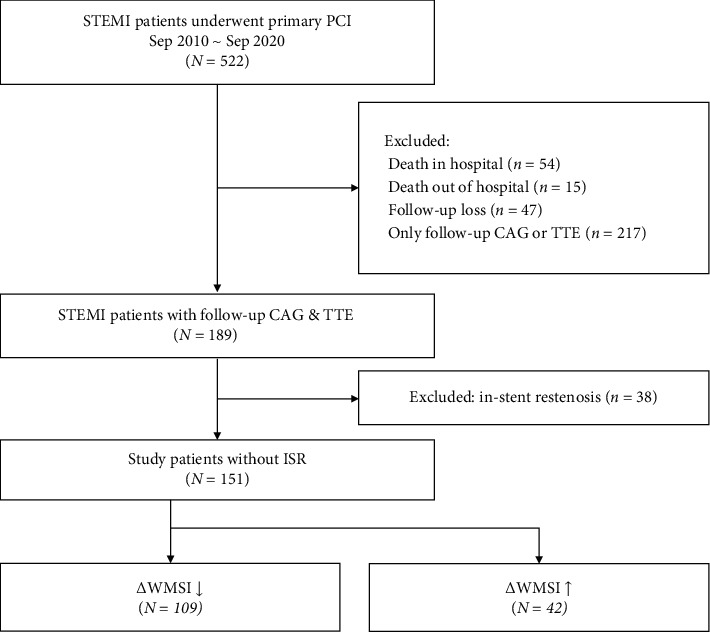
Patient flowchart. CAG = coronary angiography; ISR = in-stent restenosis; STEMI = ST-segment elevation myocardial infarction; TTE = transthoracic echocardiography; WMSI = wall motion score index.

We identified all patients with STEMI who underwent primary PCI between September 2010 and September 2020 at a single center. Among these patients, we screened patients who underwent both follow-up coronary angiography (CAG) and TTE. Patients who experienced death in or out of the hospital as well as those with follow-up loss, only CAG or TTE performed at follow-up, or in-stent restenosis (ISR) were excluded. A total of 151 patients were finally analyzed in this study (Figure 1) and were stratified as those with a decrease and those with an increase in the WMSI value during the follow-up period. This study was approved by the Kangwon National University Hospital Institutional Ethics Committee/Review Board (Study no. 2018-12-004). Informed consent was waived because of the retrospective nature of the study.

### 2.2. Clinical Data Collection

Demographic and clinical characteristics of patients with STEMI, including age, sex, medical history, Killip class, peak cardiac troponin I and creatine kinase-MB (CK-MB) isoenzyme levels, lipid profiles, and serum creatinine and hemoglobin concentrations, were recorded. Angiographic characteristics of interest included the number of diseased vessels and culprit lesions. In addition, data on medical therapies administered during hospitalization were collected.

### 2.3. STEMI and Primary PCI

STEMI was diagnosed and treated in accordance with the most recent guidelines [10, 11]. Briefly, STEMI was defined as a new ST-segment elevation of greater than 0.1 mV on at least two contiguous electrocardiographic leads or the presence of left bundle branch block in patients with acute myocardial infarction. Experienced interventional cardiologists performed primary PCI in all eligible patients with STEMI. Routine medical therapies administered during the perioperative period consisted of aspirin, P2Y12 inhibitors (e.g., clopidogrel, prasugrel, ticagrelor), heparin (either low molecular weight or conventional), and/or glycoprotein IIb/IIIa receptor antagonists. In addition, secondary preventive therapies, including statins, *β*-blockers, angiotensin-converting enzyme inhibitors, or angiotensin receptor blockers, were indicated for all patients with STEMI in the absence of contraindications.

### 2.4. TIT

The TIT was defined as the time from the onset of chest pain to the first occurrence of balloon inflation during primary PCI. This period consisted of onset-to-door and door-to-balloon times. Onset-to-door time was defined as the time from symptom onset to emergency department arrival, whereas door-to-balloon time was the time from emergency department arrival to the first instance of balloon inflation. Information on the timing of symptom onset was obtained by patient interviews. The times of emergency department arrival and first balloon inflation were obtained from patients' medical records.

### 2.5. Echocardiography and WMSI

Comprehensive TTE was performed on commercially available equipment (Vivid E9; GE Healthcare, Milwaukee, WI, USA or Acuson SC2000; Siemens Medical Solutions, Mountain View, CA, USA). Standard M-mode, two-dimensional, and color Doppler imaging were performed in parasternal, suprasternal, substernal, and apical views with positional adjustment of the patients. The initial TTE was performed within 24 hours after primary PCI. The initial and follow-up echocardiograms recorded during the study period were used to evaluate echocardiographic changes. The median interval of echocardiography was 12.3 months [interquartile range (IQR): 12.1–13.6 months]. Anatomic measurements were made according to the American Society of Echocardiography (ASE) guidelines [12]. WMSI values were obtained by dividing the LV into 16 segments from multiple short-axis views, and apical two-, four-, and long-axis views. This 16-segment model consists of six segments at both the basal and mid-ventricular levels and four segments at the apex. The attachment of the right ventricular wall to the left ventricle defines the septum, which is divided at basal and mid-left ventricular levels into anteroseptum and inferoseptum. Continuing counterclockwise, the remaining segments at both basal and mid-ventricular levels are labeled as inferior, inferolateral, anterolateral, and anterior. The apex includes septal, inferior, lateral, and anterior segments. Each segment was analyzed individually and scored on the basis of its motion and systolic thickening. Each of the segments was assigned a score based on the degree of myocardial thickening as follows: one point, normally contracting segment (or hyperkinetic segment); two points, hypokinesis; three points, akinesis; four points, dyskinesis; and five points, aneurysm. The WMSI value was calculated by dividing the sum of scores by the number of segments visualized. Left ventricular measurements and wall motion abnormalities of all patients were reviewed by 2 independent cardiologists. The observers were blinded to one another's WMSI measurements and the clinical endpoint.

### 2.6. Statistical Analysis

Continuous variables were reported as mean ± standard deviation values and were compared using Welch's *t*-test or the Wilcoxon rank-sum test, as appropriate. Categorical variables were summarized by frequencies or percentages and analyzed with the chi-squared test or Fisher's exact test, as appropriate. Changes in the WMSI value were analyzed with the paired *t*-test. Stepwise multiple linear regression analysis was used to identify the independent predictors of reductions in the WMSI value. All statistical tests were two-tailed, and *p* values of less than 0.05 were considered statistically significant. All analyses were performed using the Statistical Package for the Social Sciences version 25.0 statistical software (IBM Corporation, Armonk, NY, USA).

## 3. Results

### 3.1. Baseline Characteristics and Echocardiography

Baseline characteristics of the patients according to changes in WMSI value are summarized in Table 1. There were no significant differences in terms of age, sex, comorbidities (e.g., diabetes and hypertension), STEMI location, or medication use between patients who experienced a decrease in WMSI value and those who showed an increase in the WMSI value. The total door-to-balloon time and TIT were statistically indifferent between the two groups. However, there were more patients with TITs of 120 minutes or less among those with a decrease in the WMSI value than among those with an increase in the WMSI value (37 (34%) vs. 7 (17%); *p*=0.036). Among the laboratory findings, peak CK-MB was lower in the group of patients with WMSI value reductions. The LV end-diastolic dimension was greater in patients with a decrease in the WMSI value than those with an increase in the WMSI value (Table 2).

### 3.2. Changes in WMSI Value Based on TIT

**Figure 2:**
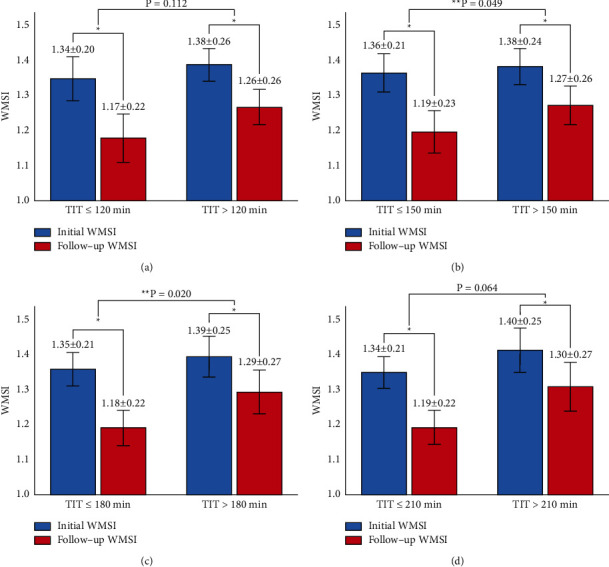
Changes in the WMSI value based on TITs of (a) 120, (b) 150, (c) 180, and (d) 210 minutes. TIT = total ischemic time; WMSI = wall motion score index.

Figure 2 shows the changes in the WMSI value according to TIT. There were no significant changes in the WMSI value between the two groups based on TITs of 120 and 210 minutes; however, WMSI values in patients with TITs of 150 minutes or less and 180 minutes or less were significantly decreased relative to those of patients with TITs of greater than 150 minutes and greater than 180 minutes (*p*=0.049 and *p*=0.020, respectively).

### 3.3. Prediction of WMSI Value Reduction in Patients with TITs of 180 Minutes or Less

In patients with TITs of 180 minutes or less, the TIT was significantly shorter among those with a reduction in the WMSI value than among those with an increase in the WMSI value (106 [81–124] vs. 133 [100–151] minutes, *p*=0.018) (Table 3). In multivariate logistic regression analysis adjusted for age, sex, body mass index, diabetes, hypertension, and smoking status, TIT was an independent predictor for a decrease in the WMSI value among these patients (adjusted hazard ratio: 0.976 (0.957–0.995); *p*=0.016) (Table 4).

## 4. Discussion

In this study, we evaluated changes in the WMSI value based on TIT in STEMI patients who underwent primary PCI and subsequent transthoracic echocardiography. The major findings of this study were as follows: (1) WMSI values in patients with TITs of 180 minutes or less were significantly decreased relative to those in patients with TITs of greater than 180 minutes during the follow-up period; (2) in patients with TITs of 180 minutes or less, the TIT was significantly shorter among the subset with a decrease in the WMSI value than those with an increase in the WMSI value; and (3) TIT was an independent predictor for recovery from regional wall motion abnormalities in patients with TITs of 180 minutes or less.

Timely performance of the primary PCI as measured by door-to-balloon time has become one of the main quality measures in the treatment of patients with STEMI [13, 14]. Several studies have failed to report improvements in mortality with shortened door-to-balloon times [15, 16]. It is possible that further reductions in the mortality rate of STEMI patients in the modern era of primary PCI and adjuvant pharmacotherapy may be achieved only by means of reducing the TIT. Minimizing TIT is a major determinant of myocardial salvage, as the prolonged duration of ischemia is related to myocardial necrosis, as evidenced by cardiac magnetic resonance (CMR) imaging [17]. Some of the new imaging techniques, such as CMR and strain, are more precise for determining myocardial damage; however, they are less accessible techniques for daily practice. Some studies have reported a good correlation between WMSI and echocardiographic strain findings [18, 19]. LV ejection fraction (LVEF) improves in some STEMI patients after effective reperfusion as a consequence of the gradual relief of myocardial stunning, whereas, in other patients, irreversible myocardial necrosis may result in chronic LV dysfunction [20]. Reverse LV remodeling occurred in a considerable proportion (37.7%) of STEMI patients who underwent primary PCI, manifesting a poor prognosis [20, 21]. The LVEF value is technique-dependent and may not accurately indicate the extent of myocardial damage due to regional compensatory effects [22–24]. On the other hand, it is estimated that an increase in the WMSI value within the first 12 to 24 hours after STEMI is a predictor of complications during hospital admission, such as malignant arrhythmia, pump failure, and mortality [25–27]. In addition, WMSI can be used to quantitatively measure the LV systolic function following STEMI [28].

Two studies reported a significant improvement in echocardiographic parameters, such as LVEF and WMSI, during follow-up after myocardial infarction [29, 30]. Such improved echocardiographic findings may indicate that well-timed and successful revascularization can restrict further myocardial remodeling. The results of follow-up echocardiography performed approximately three months after the episode suggested that, following PCI, future wall motion improvement is highly dependent on how quickly it is possible to reinstate perfusion in the occluded artery. In patients whose perfusion was restored earlier with PCI, the follow-up echocardiography reflected a significantly better degree of improvement [30]. The present study had a longer observation period than these two previous studies involving echocardiography and excluded cases of ISR through follow-up CAG; as such, we could discern the relationship between TIT and WMSI more clearly. Overall, in this study, the WMSI value tended to decrease regardless of TIT (Figure 2). However, patients who underwent primary PCI within 180 minutes experienced a more significant decrease in the WMSI value than those who underwent primary PCI after 180 minutes. This result suggests that there is an optimal TIT within which better outcomes in STEMI patients may be achieved.

In 1977, transient left circumflex coronary artery ligation was performed in dogs for different durations [31]. The percentage of transmural necrosis increased from 38% at 40 minutes of circumflex artery ligation duration to 85% at 24 hours of duration. Similar results have also been reported when evaluating cardiovascular outcomes in STEMI patients receiving fibrinolysis. For every 1,000 patients, 15 more lives were saved at one month of follow-up if the patients received treatment an hour earlier in the European Myocardial Infarction Project Group [32]. A meta-analysis of 22 randomized controlled trials studied more than 50,000 STEMI patients who received fibrinolysis. If fibrinolysis was achieved within one hour of symptom onset, there were 65 fewer deaths for every 1,000 patients when compared to patients who experienced greater delays from symptom onset to fibrinolysis [33]. Both animal and clinical data support the notion that shortening the duration of infarct artery occlusions can lead to smaller infarct sizes and lower mortality rates. However, the relationship is not linear and most of the benefit of reperfusion is seen within the first two hours of occlusion. In the CMR study, patients with a symptom-to-balloon time of greater than 121 minutes had significantly greater transmural necrosis, a larger infarct size, and decreased myocardial salvage [34]. The current guidelines recommend primary PCI as the preferred reperfusion strategy over thrombolysis in patients with STEMI, provided it can be performed within 120 minutes from STEMI diagnosis [10]. This would correspond with prolonged symptom-onset-to-balloon times of 3 to 4 hours. Of 1,791 patients with STEMI treated by primary angioplasty, a symptom-onset-to-balloon time of more than four hours was an independent predictor of one-year mortality [35]. In the Acute Coronary Syndrome Israeli Survey registry (involving 2,254 patients with STEMI treated by primary PCI), shortening of the TIT to less than 150 minutes was associated with improved long-term survival rates [36]. In the current study, based on a TIT of 180 minutes, a significant reduction in the WMSI value was experienced within a median follow-up period of 1 year. This result may explain why the optimal TIT exceeds two hours in clinical studies conducted in the modern reperfusion era of STEMI. There were significant changes in the WMSI value based on TITs of 150 and 180 minutes; especially considering a TIT of 180 minutes, TIT was an independent predictor of recovery from regional wall motion abnormalities. This study is meaningful in suggesting the criteria for the optimal TIT based on the WMSI value in STEMI patients.

### 4.1. Limitations

There are several limitations to this study. First, this was a retrospective study that was performed at a single academic hospital involving a relatively small sample population. However, all patients underwent follow-up CAG and had no ISR identified. In addition, this study had a relatively long observation period for recovery from regional wall motion abnormalities. Second, reporting bias is an inherent limitation of studying symptom‐to‐balloon time. Patients may find it difficult to accurately estimate the time of symptom onset, especially in the acute setting when suffering from pain and stress. Therefore, symptom‐to‐balloon times may not have been independently verified.

## 5. Conclusion

In the modern reperfusion era of STEMI, patients with TITs of 180 minutes or less showed a significant degree of recovery from regional wall motion abnormalities compared to those with TITs of greater than 180 minutes. Every possible effort should be made to reduce TIT to 180 minutes or less, and further treatment for patients with TITs of greater than 180 minutes should be considered.

## Figures and Tables

**Table 1:** Baseline characteristics.

	DWMSI↓ (*n* = 109)	DWMSI↑ (*n* = 42)	*p* value
Demographics			
Age, years	62 ± 13	61 ± 10	0.565
Male	82 (75)	33 (79)	0.666
Body mass index, kg/m^2^	24.1 ± 3.0	24.5 ± 3.0	0.484
Medical history			
Diabetes	36 (33)	12 (29)	0.598
Hypertension	52 (48)	24 (57)	0.299
Smoking ever	52 (48)	21 (50)	0.800
Previous stroke	6 (5.5)	1 (2.4)	0.413
Previous PCI	7 (6.4)	0 (0.0)	0.093
Clinical presentation			
Preinfarction angina	7 (6.4)	4 (9.5)	0.511
STEMI location			
Anterior	57 (52)	13 (31)	0.075
Inferior	44 (40)	24 (57)	
Lateral	5 (4.6)	5 (12)	
Posterior	2 (1.8)	0 (0.0)	
Killip class			
I	74 (68)	32 (76)	0.521
II	17 (16)	7 (17)	
III	5 (4.6)	1 (2.4)	
IV	13 (12)	2 (4.8)	
Culprit vessels			
Left main coronary artery	1 (0.9)	0 (0.0)	0.010
Left anterior descending artery	69 (63)	15 (36)	
Left circumflex artery	5 (4.6)	6 (14)	
Right coronary artery	34 (31)	21 (50)	
Extent of CAD			
1VD	86 (79)	38 (91)	0.222
2VD	20 (18)	3 (7.1)	
3VD	3 (2.8)	1 (2.4)	
TIMI flow			
Grade 2	10 (9.2)	4 (9.5)	0.947
Grade 3	99 (91)	38 (90)	
De novo lesions at follow-up CAG	16 (15)	4 (9.5)	0.402
Door-to-balloon time, minutes	49 (38–62)	48 (36–59)	0.453
Total ischemic time, minutes	178 (105–368)	204 (133–372)	0.232
Group according to total ischemic time (TIT)			
≤120 minutes	37 (34)	7 (17)	**0.036**
120–150 minutes	10 (9.2)	7 (17)	0.192
150–180 minutes	8 (7.3)	5 (12)	0.370
180–210 minutes	7 (6.4)	4 (9.5)	0.511
>210 minutes	47 (43)	19 (45)	0.814
Medications			
Aspirin	109 (100)	42 (100)	—
Clopidogrel	92 (84)	31 (74)	0.133
Ticagrelor	4 (3.7)	4 (9.5)	0.150
Prasugrel	14 (13)	8 (19)	0.333
Cilostazol	19 (17)	8 (19)	0.816
Statin	109 (100)	42 (100)	—
*β*-Blockers	93 (85)	37 (88)	0.659
ACE inhibitor	24 (22)	11 (26)	0.586
Angiotensin II receptor blocker	57 (52)	19 (45)	0.437
Laboratory assessments			
Hemoglobin, g/dL	14.4 ± 2.1	14.3 ± 2.4	0.759
CRP, mg/dL	0.99 ± 3.0	0.55 ± 1.5	0.370
BUN, g/dL	15.4 ± 5.3	14.8 ± 5.8	0.521
Creatinine, g/dL	0.89 ± 0.25	0.88 ± 0.28	0.785
Glucose, mg/dL	173 ± 79	161 ± 48	0.346
HbA1c, %	6.8 ± 1.8	6.4 ± 1.2	0.171
Total cholesterol, mg/dL	177 ± 45	180 ± 50	0.723
LDL, mg/dL	118 ± 49	116 ± 51	0.860
CK-MB, ng/mL	147 ± 110	213 ± 109	**0.001**
Troponin I, pg/mL	30.4 ± 34.3	34.8 ± 21.3	0.440
BNP, pg/mL	104 ± 199	138 ± 209	0.382

Values are presented as mean ± standard deviation, *n* (%), or median (interquartile range). ACE, angiotensin-converting enzyme; BNP, brain natriuretic peptide; BUN, blood urea nitrogen; CAD, coronary artery disease; CAG, coronary angiography; CK-MB, creatine kinase-MB fraction; CRP, C-reactive protein; HbA1c, hemoglobin A1c; LDL, low-density lipoprotein; PCI, percutaneous coronary intervention; STEMI, ST-segment elevation myocardial infarction; TIMI, thrombolysis in myocardial infarction. Statistical significance was defined as *p* < 0.05 by Welch's *t*-test (continuous variables) or the chi-squared test (categorical variables). The values in bold indicate statistical significance (*p* < 0.05).

**Table 2:** Echocardiographic parameters.

	ΔWMSI↓ (*n* = 109)	ΔWMSI↑ (*n* = 42)	*p* value
LV end-diastolic dimension, mm	48.2 ± 4.1	50.0 ± 4.8	**0.027**
LV end-systolic dimension, mm	32.3 ± 4.5	34.0 ± 6.3	0.064
Interventricular septum thickness, mm	9.7 ± 1.4	9.8 ± 1.1	0.700
LV posterior wall thickness, mm	9.6 ± 1.2	9.9 ± 1.2	0.256
LV ejection fraction, %	51.9 ± 8.4	52.5 ± 8.7	0.669
LA volume index, ml/m^2^	33.4 ± 11.4	33.9 ± 9.0	0.812
Early diastolic mitral inflow velocity (E), m/s	0.59 ± 0.19	0.65 ± 0.20	0.101
Late diastolic mitral inflow velocity (A), m/s	0.74 ± 0.18	0.76 ± 0.21	0.465
Mitral annulus early diastolic velocity (e′), m/s	0.06 ± 0.02	0.07 ± 0.07	0.477
E/*e*′	10.8 ± 5.0	12.6 ± 5.9	0.064
RV systolic pressure, mm Hg	25.5 ± 8.5	25.8 ± 10.1	0.840

Values are presented as mean ± standard deviation. LA, left atrium; LV, left ventricle; RV, right ventricle. Statistical significance was defined as *p* < 0.05 by Welch's *t*-test (continuous variables). The value in bold indicates statistical significance (*p* < 0.05).

**Table 3:** Variables associated with a decrease in the WMSI value among patients with TITs of 180 minutes or less (*N* = 74).

	ΔWMSI↓(*n* = 55)	ΔWMSI↑(*n* = 19)	*p* value
Age	61 ± 14	61 ± 11	0.971
Male sex	44 (80)	14 (74)	0.564
Body mass index	24.2 ± 2.8	25.2 ± 3.1	0.188
Diabetes	14 (26)	7 (37)	0.343
Hypertension	27 (49)	12 (63)	0.290
Smoking ever	26 (47)	6 (32)	0.234
STEMI, anterior	29 (53)	7 (37)	0.232
Total ischemic time, minutes	106 (81–124)	133 (100–151)	**0.018**
CK-MB	134 ± 103	168 ± 113	0.244
LV end-diastolic dimension, mm	47.9 ± 3.9	49.1 ± 5.7	0.301

Values are presented as mean ± standard deviation, *n* (%), or median (interquartile range). CK-MB, creatine kinase-MB fraction; LV, left ventricle; STEMI, ST-segment elevation myocardial infarction. Statistical significance was defined as *p* < 0.05 by Welch's *t*-test (continuous variables) or the chi-squared test (categorical variables). The values in bold indicate statistical significance (*p* < 0.05).

**Table 4:** Predictor of a decrease in the WMSI value among patients with TITs of 180 minutes or less.

	Univariate		^*∗*^Multivariate	
	OR (95% CI)	*p* value	OR (95% CI)	*p* value
Total ischemic time, minutes	0.980 (0.964–0.997)	0.023	0.976 (0.957–0.995)	0.016

^*∗*^Adjusted for age, sex, body mass index, diabetes, hypertension, and smoking status.

## Data Availability

The data used to support the findings of this study are available from the corresponding author upon reasonable request.

## Abbreviations

CMR: Cardiac magnetic resonance
LV: Left ventricle
PCI: Percutaneous coronary intervention
STEMI: ST-segment elevation myocardial infarction
TIT: Total ischemic time
WMSI: Wall motion score index.
